# Standardized Manual-Based Smoking Cessation Treatment in Patients With Comorbid Mental Disorders: Qualitative Interview Study

**DOI:** 10.2196/97001

**Published:** 2026-09-23

**Authors:** Johanna Bück, Esra Sünkel, Alla Machulska, Marie Neubert, Tim Klucken

**Affiliations:** 1 Clinical Psychology and Psychotherapy, Department of Psychology, University of Siegen, Obergraben 23, Siegen, North Rhine-Westphalia, 57072, Germany, 49 271-740 ext 4140

**Keywords:** smoking cessation, mental health, barriers, facilitators, guideline-conform treatment, thematic analysis

## Abstract

**Background:**

Smoking prevalence is disproportionately high among individuals with mental disorders, who also show reduced cessation success, suggesting that current cessation interventions may not fully address their specific needs. A barrier to smoking cessation is the fear that quitting may worsen mental health, yet little is known about how individuals with mental disorders experience guideline-conform cessation treatments, which may address such concerns.

**Objective:**

This qualitative study explored the perspectives of smoking individuals with comorbid mental disorders who participated in a standardized, manual-based, cognitive behavioral smoking cessation program to identify targets for optimizing guideline-conform interventions for this population, spanning treatment engagement, perceived challenges, and facilitators within this context.

**Methods:**

Semistructured interviews were conducted with 11 participants representing diverse cessation trajectories (sustained abstinence, relapse, and no quit attempt) 6 months after treatment completion. Data were analyzed using reflexive thematic analysis.

**Results:**

Three overarching themes were identified. First, participants described smoking cessation as a multidimensional, processual experience extending beyond behavioral change to encompass psychological and social dimensions, characterized by nonlinearity, adaptive meaning-making, and improvements in self-efficacy despite transient strain during early abstinence. Second, smoking was experienced as a functionally embedded practice serving affective, habitual, and social functions, with motivation emerging as dynamic and relationally sustained rather than stable. Third, treatment engagement was shaped by motivational states and contextual circumstances, with participants offering favorable evaluations of the structured intervention alongside recommendations for greater individualization and flexibility. Notably, mental health–related concerns about cessation largely persisted despite predominantly positive or neutral mental health outcomes, a pattern consistent with cognitive immunization processes.

**Conclusions:**

Guideline-conform smoking cessation treatment appears feasible and largely well-received by individuals with comorbid mental disorders. The findings highlight the need for targeted psychoeducation to address persistent misconceptions about the mental health risks of cessation, as well as individualized, context-sensitive treatment adaptations, including greater flexibility in session structure, motivational support beyond the active treatment phase, and explicit attention to the social and functional embeddedness of smoking, to improve cessation outcomes in this high-risk population.

## Introduction

Tobacco use constitutes one of the leading causes of premature mortality and morbidity [1], due to its association with a wide range of adverse health outcomes, including cancer [2], chronic respiratory illness [3], and the development of cardiovascular disease [4]. Smoking is the most prevalent form of tobacco consumption [5], with more than one billion individuals estimated to smoke globally [6]. Notably, smoking prevalence is markedly elevated among individuals with mental disorders, who smoke at rates at least twice as high as those observed in the general population [7,8]. This disparity is particularly concerning, given that smoking is estimated to account for up to two-thirds of the reduced life expectancy observed among people with mental disorders compared to those without [9]. The elevated prevalence of smoking in this population has been linked to increased vulnerability to initiate smoking, often as a means of regulating negative affect [10]. Although smoking may provide short-term affect regulation, accumulating evidence indicates that long-term smoking is associated with detrimental effects on mental health. Specifically, smoking has been linked to decreases in mental well-being [11], heightened stress levels [12,13], and a progressive exacerbation of symptom severity in mental disorders. These adverse effects are thought to arise from several interacting mechanisms, including recurrent experiences of nicotine withdrawal [14] and attenuated responsiveness of the mesolimbic reward system [15-17]. Together, these findings point to a bidirectional relationship between smoking and mental health [18-21].

Compounding this burden, individuals affected by mental disorders are less likely to achieve sustained nicotine cessation compared to smokers without such conditions [8]. While relapse rates following unaided quit attempts reach up to 95%, even in the general population [22], guideline-conform cessation treatments substantially increase the likelihood of successful cessation [23]. Such guideline-oriented approaches are commonly grounded in cognitive behavioral principles, accompanied by nicotine replacement products (ie, nicotine gum) if patients exhibit strong nicotine dependence [7]. Nevertheless, even when receiving these treatments, individuals with mental disorders exhibit disproportionately high relapse rates [24]. These findings suggest that existing smoking cessation treatments may not be sufficiently tailored to the specific needs of individuals with mental disorders. Identifying which aspects of existing guideline-conform smoking cessation treatments are perceived as supportive and which may pose additional challenges for this population is therefore crucial for informing targeted improvements.

In this context, qualitative research methods are particularly well suited to capturing the complexity of treatment needs and lived experiences of smoking individuals with mental disorders. Smoking behavior is closely intertwined with everyday routines, social interactions, and individual meaning-making processes [25], which are not readily accessible through structured quantitative assessments alone. Accordingly, a small number of studies have used qualitative approaches to evaluate smoking cessation interventions for individuals with severe mental illness like schizophrenia or bipolar disorder [26], which demonstrate that tailored cessation treatments are perceived as more beneficial than generic smoking cessation services due to their higher flexibility and personalization. However, they provide limited insight into how standardized guideline-conform smoking cessation treatments might need to be adapted to better meet the needs of individuals with common mental disorders.

Indications of these needs can be found in qualitative studies that have examined perceived facilitators and barriers to smoking cessation among individuals with mental disorders *outside* the context of structured cessation programs. For instance, prior studies have explored the experiences of individuals with self-reported depression in general practice–based settings [27] and pregnant women with mental disorders who smoke [28]. These studies suggest that the use of smoking as a means of coping with acute distress functions as a key psychological barrier to smoking cessation among individuals with common mental disorders [27,28]. Importantly, they also demonstrate that this reliance on smoking as a coping strategy gives rise to a pronounced desire for tailored and professionally supported cessation interventions, especially with respect to psychological care [27]. Crucially, these treatment-related needs extend beyond structural or procedural aspects of cessation support and are closely linked to mental health-specific beliefs and concerns about quitting. Individuals with mental disorders often fear a deterioration of their mental well-being following cessation [29]. Notably, these concerns are frequently shared by health care professionals [30-32], despite a substantial body of evidence demonstrating that smoking cessation is associated with pronounced improvements in mental health [33-35]. Importantly, these concerns pose a substantial barrier to cessation attempts outside structured treatment programs [36,37]. However, it remains unclear to what extent barriers identified in such cessation contexts also operate within standardized, guideline-conform cessation programs, or whether such structured interventions mitigate, transform, or introduce distinct challenges for smokers with common mental disorders. Unlike cessation outside of unstructured cessation treatments, guideline-conform programs typically incorporate structured psychoeducation and behavioral strategies from cognitive behavioral approaches [38,39], which may explicitly address mental health–related concerns and coping expectations associated with smoking cessation. At the same time, their standardized and time-limited nature may also generate novel challenges, for example, through fixed treatment schedules, standardized content, or insufficient flexibility to accommodate individual mental health needs.

To date, patients’ lived experiences in the context of such structured interventions have not been systematically explored, with most qualitative research focusing on cessation outside formal programs or on highly tailored treatments for severe mental illness. Investigating these experiences may therefore clarify how treatment components are perceived, which aspects support or hinder engagement, and how guideline-conform cessation treatment can be improved to support sustained abstinence.

To this end, the present study aimed to explore the experiences of individuals with comorbid mental disorders participating in a manual-based, guideline-conform smoking cessation treatment [38,39], using semistructured interviews to capture nuanced insights into treatment engagement, perceived challenges, and facilitators within this context.

## Methods

### Study Design

The present study used a qualitative design using semistructured interviews adapted from the Client Change Interview (CCI) [40] in a face-to-face setting. Data were analyzed using thematic analysis [41], as this method allows for a detailed and nuanced account of experiences while remaining highly flexible [41]. The qualitative component was embedded within 2 randomized controlled trials (RCTs) [40,41] evaluating a standardized cognitive behavioral smoking cessation intervention for individuals with mental disorders compared with a waitlist control. One RCT examined an intervention among individuals awaiting outpatient psychotherapy, while the other evaluated the intervention as an adjunct to ongoing outpatient psychotherapy. For full trial details, see [42,43].

### Ethical Considerations

The qualitative follow-up study was conducted in accordance with the Declaration of Helsinki and approved by the Ethics Committee of the University of Siegen (ER_10_2024). Written informed consent was obtained from all participants prior to conducting the interviews. Participants were informed that their data would be used for research purposes and that participation was voluntary. Data were deidentified through the use of participant codes (eg, P01 and P02) to protect confidentiality; no personally identifiable information is reported in the manuscript. No financial compensation was offered to participants.

### Participants

The participants consisted of 11 smoking individuals with comorbid mental disorders recruited from the 2 RCTs (awaiting psychotherapy: n=6; adjunctive to psychotherapy: n=5). Inclusion criteria for the RCTs were (1) dependent use of tobacco according to the *ICD-10* (*International Statistical Classification of Diseases, Tenth Revision*), diagnostic criteria for tobacco dependence (F17.2); (2) meeting ICD-10 diagnostic criteria for a primary mental disorder; (3) being aged ≥18 years; and (4) German language skills. Participation in the interviews required completion of the smoking cessation treatment provided within each RCT. Exclusion criteria comprised severe cognitive impairment and acute suicidality. The sample included individuals who had successfully quit smoking (n=4), individuals who had relapsed after a cessation attempt within the treatment (n=5), and individuals who had not attempted smoking cessation (n=2), thereby capturing a broad range of treatment experiences.

### Procedure

#### Data Collection

Data collection for the present qualitative study took place between April and October of 2025. The RCT procedures are summarized briefly to provide contextual background for the qualitative follow-up study, which constitutes the primary focus of the present study. In the RCTs, interested individuals were initially contacted for a telephone screening interview to assess eligibility (*t*_−1_). Eligible participants were then invited to the Psychotherapeutic Outpatient Center of the University of Siegen, where written informed consent was obtained and baseline assessments were conducted as part of a comprehensive diagnostic session (*t*_₀_). Following baseline assessment, participants were randomly assigned to either the experimental or a waitlist control condition. Participants in the experimental condition received a manual-based, 6-session smoking cessation intervention over a 3-week period, complemented by access to a smartphone-based self-help application. Postintervention assessments were conducted after treatment completion (*t*_₁_), with follow-up assessments at 6 weeks (*t*_₂_) and 6 months (*t*_₃_). Participants enrolled in the second RCT continued their ongoing outpatient psychotherapy throughout the study period.

#### Cognitive Behavioral Smoking Cessation Intervention

The intervention in the experimental group consisted of a manual-based, 6-step smoking cessation program that was adapted from a continuously evaluated tobacco cessation program in Germany, developed by the Smoking Cessation Working Group (Arbeitskreis Tabakentwöhnung) at the University Hospital for Psychiatry and Psychotherapy in Tübingen. The program, outlined in the manual by Batra and Buchkremer [38,39], is grounded in principles of cognitive behavioral therapy (CBT) and can be delivered in both group and individual settings. In the present study, the treatment was implemented individually. Over the course of 6 structured sessions, participants were guided through preparation and initiation of smoking cessation, the development of strategies to maintain abstinence, and relapse prevention techniques. At each session, carbon monoxide (CO) levels in exhaled breath were measured as part of the intervention using the Smokerlyzer. The sessions were organized into 3 phases (preparation, cessation, and maintenance), each addressing specific cognitive and behavioral aspects of quitting. Personalized recommendations were provided to tailor the treatment to each participant’s smoking history and psychological needs. In this research project, the original program was intensified to meet the needs of smokers with mental disorders by increasing the frequency of sessions and integrating a digital self-help component. Whereas the initial program was designed with weekly sessions, the intensified version comprised 2 sessions per week, resulting in a total intervention duration of 3 weeks. The digital self-help component consisted of access to the digital health application NichtraucherHelden (Sanero Medical GmbH). This CBT-based application comprises 8 interactive modules addressing psychoeducation, motivational enhancement, coping strategies, behavioral exercises, and relapse prevention. The application was designed to complement the face-to-face intervention by facilitating the consolidation of skills between sessions. A recent nationwide, multicenter RCT demonstrated the feasibility and efficacy of this guideline-based smoking cessation app, with approximately doubled 7-day point prevalence abstinence rates compared with a minimal-intervention control condition (20.2% vs 10.5%), which consisted of a brief 3-minute advice session [44].

#### Half-Standardized Interview Procedure

The qualitative follow-up study drew on the RCT samples outlined above. All participants who had completed the smoking cessation intervention in the treatment arms of the 2 RCTs and whose 6-month follow-up appointments fell within the interview data-collection period were contacted by telephone and invited to participate in the face-to-face interviews, which were conducted in a 1:1 setting in the Psychotherapeutic Outpatient Center of the University of Siegen without any other individuals present. The target sample size was informed by methodological recommendations for qualitative interview studies, which suggest that 6‐12 participants are sufficient to obtain in-depth insights [45]. Consistent with Braun and Clarke’s [46] recommendations regarding sample size rationales in reflexive thematic analysis, sample adequacy was evaluated in terms of the depth, richness, and internal coherence of the dataset as a whole, reflecting our understanding that in a constructionist analytic approach themes are researcher constructions rather than entities residing in the data. It was nonetheless noted during analysis that later transcripts contributed progressively fewer new codes relative to earlier ones, which was taken as an empirical indication of sufficient dataset breadth for addressing the study’s research questions, without implying that the analytic construction of themes was complete or exhaustive. Eleven participants consented to participate and were included in the present analyses, while 6 participants declined participation in the interviews. The qualitative interviews were conducted in German by JB, a trained clinical psychologist, 6 months after completion of the smoking cessation intervention (*t*_₃_). Interview duration ranged from 8 to 26 minutes (mean 17.27, SD 5.76 minutes). The interviews commenced with the interviewer posing the open-ended question: “How did you perceive the smoking cessation treatment in general?” This inquiry enabled participants to expound on their experience with the smoking cessation treatment in a free and spontaneous manner. Subsequent to the initial open-ended question, a semistructured interview procedure, adapted from the CCI [40], was carried out. The original CCI was developed to explore patient perspectives on psychotherapy, with a focus on perceived facilitators and barriers to change, attribution of experienced changes, and perceived missing elements in treatment. The interview guide is provided in Multimedia Appendix 1. For the present study, the interview guide was adapted to ensure relevance to smoking cessation while preserving its inductive character. Specifically, questions were phrased in an open-ended manner and tailored to address experiences with the smoking cessation treatment, study participation, and perceived psychological and social consequences of smoking cessation, without prespecifying response options or hypotheses. Hence, although the interview guide provided a loose structure by delineating broad thematic areas, the interview approach remained intentionally open and exploratory. Participants were encouraged to elaborate freely, determine the order and emphasis of topics, and introduce new themes beyond the predefined prompts.

All interviews were audio-recorded and transcribed verbatim using the automatic transcription function of f4x. Transcripts were subsequently reviewed by the authors to ensure accuracy.

### Data Analysis

For the evaluation of the interview material, a reflexive thematic analysis was performed following the methodological framework proposed by Braun and Clarke [41,47]. The analytic approach was inductive, with themes generated from participants’ accounts rather than from predefined theoretical constructs or hypotheses. Although the semistructured interview guide delineated broad areas of inquiry, it did not determine the analytic outcomes. Instead, analysis focused on identifying patterns of meaning across the dataset through iterative engagement with the data. All 11 transcripts were imported into MAXQDA (version 2024) for systematic data management and analysis. Initial coding was data-driven and conducted independently by two trained clinical psychologists (JB and ES), who systematically examined all 11 transcripts and generated preliminary codes grounded in participants’ narratives.

Following independent coding, the analysts met to discuss, compare, and refine codes through an iterative process of critical reflection. Codes were progressively clustered and organized into subthemes and overarching themes through collaborative discussion, with the hierarchical structure reviewed and refined recursively against the dataset until a coherent and grounded analytic narrative emerged. In line with a reflexive approach, emphasis was placed on interpretative sense-making rather than on interrater reliability*,* with themes understood as analytic constructions shaped through sustained engagement with the data.

### Reflexivity and Positionality

Both analysts brought relevant professional backgrounds to the analytic process that warrant explicit acknowledgment. JB, who conducted the interviews and contributed to the analysis, is a female trained clinical psychologist (MSc) who completed a training for the manual-based program used as the intervention in the present study. JB had no contact with the participants prior to conducting the interviews. ES, the second analyst, is likewise a female trained clinical psychologist and psychotherapist-in-training in CBT, who also completed the training for the manual-based intervention. These backgrounds may have introduced a degree of familiarity with the intervention that could have influenced the analytic process, for instance, by shaping sensitivity to certain participant experiences or generating implicit assumptions about treatment processes. To mitigate this, the analytic approach was deliberately kept inductive and data-driven, themes were constructed through independent coding followed by critical discussion between both analysts, and interpretations were continuously grounded in participants’ own accounts rather than in the analysts’ prior knowledge of the intervention.

### Trustworthiness

The trustworthiness of the findings was addressed in line with Lincoln and Guba’s [48] criteria for qualitative research. Credibility was supported by independent coding by two trained researchers followed by iterative consensus-based discussion, verbatim grounding of interpretations in participant quotations, and prolonged engagement with the sample through longitudinal follow-up appointments within the broader RCT. Dependability was ensured through verbatim transcription, systematic data management using MAXQDA (version 2024), a documented analytic procedure, and the reflexivity statement above. Confirmability was addressed through the inductive analytic approach and the 2-author independent coding process, which served as a check on individual interpretive bias. Transferability is necessarily limited by the characteristics of the present sample, as discussed in the Limitations section; however, thick description and extensive verbatim quotations throughout the Results section allow readers to evaluate the relevance of findings to their own contexts.

The reporting of the present study followed the COREQ (Consolidated Criteria for Reporting Qualitative Research) checklist [49], and a completed COREQ checklist is provided as Checklist 1.

## Results

### Participants’ Characteristics

In total, 11 individuals (5 female, 6 male; mean age 30.81, SD 9.98 years) agreed to participate. Table 1 provides a comprehensive overview of the interviewees’ characteristics. Nine participants had a diagnosed recurrent depressive disorder, 1 participant was diagnosed with a social anxiety disorder, and 1 participant with bulimia nervosa. At the time of the interview, 4 participants had maintained smoking abstinence. Five participants reported having made at least one cessation attempt between treatment initiation and the interview but had subsequently relapsed. Two participants reported no cessation attempt within the treatment initiation and the time of the interview.

**Table 1.** Participants’ characteristics.

Patient code	Age (years)	Sex	Primary diagnosis (ICD-10^a^)	Smoking status at time of interview
P01	20‐39	Female	Social anxiety disorder	Sustained abstinence
P02	20‐39	Female	Recurrent depressive disorder	Relapse following cessation attempt
P03	20‐39	Female	Recurrent depressive disorder	Relapse following cessation attempt
P04	20‐39	Female	Recurrent depressive disorder	Sustained abstinence
P05	>40	Male	Recurrent depressive disorder	Sustained abstinence
P06	20‐39	Male	Recurrent depressive disorder	Sustained abstinence
P07	20‐39	Female	Bulimia nervosa	Relapse following cessation attempt
P08	20‐39	Male	Recurrent depressive disorder	Relapse following cessation attempt
P09	20‐39	Male	Recurrent depressive disorder	Relapse following cessation attempt
P10	20‐39	Male	Recurrent depressive disorder	No cessation attempt
P11	>40	Male	Recurrent depressive disorder	No cessation attempt

a *ICD-10*: *International Statistical Classification of Diseases, Tenth Revision*.

### Overview of the Thematic Analysis Framework

All interviews were conducted in German. Quotations are presented as literal English translations and appear indented and italicized in the respective sections. The analysis identified 3 overarching themes: *outcomes of the smoking cessation treatment*, *facilitating and impeding factors*, and *perceptions of the treatment*. Table 2 provides an overview of all themes and subthemes and their specific analytical focus identified in the analysis process. The analytical focus indicates the perspective through which the data were interpreted, thus clarifying the specific analytic questions each theme addresses within the overall analysis.

**Table 2.** Overview of overarching themes and subthemes identified in the reflexive thematic analysis.

Overarching theme	Subthemes	Analytical focus
Smoking cessation as a multidimensional, processual experience	Smoking-related changes Changes in mental well-being Personal and social consequences of cessation as contextual influences on abstinence maintenance	Consequences and experiential effects
Smoking as functionally embedded: personal, relational, and contextual barriers and facilitators	Perceived functionality and automaticity of smoking Motivation Social and contextual influences Indication	Processes shaping cessation trajectories
Treatment engagement as contextually and motivationally contingent	Manual-based intervention App-based treatment Perceived mismatch between treatment structure and individual needs	Evaluation and optimization of intervention

### Theme 1: Smoking Cessation as a Multidimensional, Processual Experience

Participants described smoking cessation as a complex, nonlinear process extending beyond changes in smoking behavior to encompass psychological, personal, and social dimensions. Rather than reflecting discrete endpoints of success or failure, participants’ accounts revealed dynamic trajectories shaped by shifting practices, emotional fluctuations, and evolving personal resources.

#### Subtheme 1: Smoking-Related Changes

Participants reported varying degrees of cessation success. Several participants described successful cessation even under conditions of substantial psychological distress or long-standing smoking histories:

I’ve managed to do that [quit smoking] for now. I smoked for 13 years, 14 years, which is ages, since I was 14. And, um, yes, of course, it’s a big deal.

[Participant 9, position 34]

Beyond sustained cessation, several accounts highlighted the nonlinear nature of behavior change, describing initial success followed by relapse, or difficulties maintaining reduced consumption over time:

[…] and I did try to be a bit more conscious about limiting my consumption, which worked well at first. But then, over time, I fell back into my old pattern.

[Participant 10, position 2]

Across these outcome patterns, participants frequently reported reductions in craving and increased awareness of situational smoking cues, indicating enhanced self-monitoring. Two participants described increased smoking, which was attributed primarily to changes in nicotine delivery methods, rather than a perceived failure of cessation efforts:

Well, I would say that I actually smoke more overall because I no longer smoke normal cigarettes, but these Heets [heated tobacco sticks], i.e. IQOS [a heated tobacco product system by Philip Morris International]. And […] it doesn’t satisfy me as much as a normal cigarette.

[Participant 3, position 6]

#### Subtheme 2: Changes in Mental Well-Being

Changes in smoking behavior were closely linked to perceived alterations in mental well-being. For instance, participants frequently reported increases in positive affect, energy, and overall mood following abstinence or reduced smoking. These psychological improvements were predominantly attributed to experiences of mastery and self-efficacy:

So, when I managed […] to stop again, I was proud of myself and I had this feeling of, okay, I can do this, and I experienced self-efficacy.

[Participant 3, position 34]

Other participants ascribed psychological changes to improved stress regulation through newly acquired coping strategies, such as relaxation techniques, which were also incorporated into the cessation treatment:

We did a relaxation exercise like that once, and it definitely helped me, because I sometimes do it a little bit at home now. And it definitely helps me to be a little more relaxed in general.

[Participant 7, position 56]

Social validation further reinforced positive psychological outcomes:

So, the first month, when I actually stopped smoking, was really good. There were some really great days, and I got a lot of positive feedback from my peer group.

[Participant 9, position 32]

Participants also described significant psychological strain during early abstinence:

Wow, the insomnia, it’s so bad [...] The first three days, that was really something. I was pissed off. […] It was wild.

[Participant 9, position 50]

However, these adverse experiences were predominantly framed as temporally bound. Even when withdrawal symptoms persisted beyond the anticipated 2-week period, participants described them as a necessary transitional phase preceding improvement.

But I have to say, it’s also true that something really important is missing at first, and you just have to go through a certain phase that’s kind of uncomfortable before things get better.

[Participant 3, position 34]

Moreover, similar to withdrawal-related distress, negative affect following relapse was also perceived as transient:

When I started again, I actually felt bad after the first few cigarettes. I felt a little guilty that I couldn’t stick with it. But then it was over relatively quickly for me.

[Participant 2, position 84]

#### Subtheme 3: Personal and Social Consequences of Cessation as Contextual Influences on Abstinence Maintenance

Beyond changes in smoking behavior and mental well-being, participants described a range of personal and social consequences following cessation or reduction that shaped their ongoing motivation and capacity to maintain abstinence. These consequences were not experienced as neutral outcomes but as contextual factors that either reinforced or complicated sustained engagement with the cessation process. Gains in physical health, time, and financial resources were frequently described as tangible reinforcers of continued abstinence:

It also makes sport easier. That’s something that’s good for your mental health too, when you can cycle up a hill without being completely exhausted.

[Participant 5, position 10]

Conversely, participants reported social losses associated with reduced smoking. In some cases, individuals described consciously distancing themselves from smoking-related social contexts to protect their cessation efforts:

At first, I was a little afraid to make contact, […] that I wouldn’t be able to distance myself from it [the smoking].

[Participant 1, position 26]

Such distancing was perceived differently, with participants reporting positive and negative experiences:

It was all gone in one fell swoop. That social bonding. The cigarette.

[Participant 8, position 93]

This ambivalence was especially pronounced in close relationships. One participant described reduced contact with a family member in the absence of shared smoking moments:

With my father […] I just notice that I can build a better relationship with him or maintain it when we share these moments of smoking. […] I also notice that when I don’t smoke, […] we don’t talk as much anymore.

[Participant 3, position 80]

In sum, participants reported heterogeneous outcomes of the smoking cessation treatment within this overarching theme. While many described sustained improvements in smoking behavior and mental well-being, others experienced temporary declines in mental well-being due to withdrawal or relapse. Importantly, personal and social consequences of cessation emerged not merely as peripheral outcomes but as contextual factors shaping ongoing motivation and the capacity to maintain abstinence, underscoring the need for interventions that explicitly address the social embeddedness of smoking and support individuals in navigating the personal costs of cessation.

### Theme 2: Smoking as Functionally Embedded: Personal, Relational, and Contextual Barriers and Facilitators

Participants’ accounts highlighted that smoking was deeply embedded in affective, habitual, and social functions, which constituted both the primary barriers to cessation and the central targets of change. Facilitators and impediments to cessation were thus not experienced as isolated factors but as expressions of smoking’s multifaceted role in everyday life.

#### Subtheme 1: Perceived Functionality and Automaticity of Smoking

When describing facilitators and barriers to smoking cessation, participants frequently referred to the affect-regulatory, rewarding (“…that it’s such a pleasant moment for me in the evening, a bit like a reward” [Participant 3, position 14]), and instrumental functions smoking served in their everyday lives. Additionally, smoking embedded in habitual routines and automatic behaviors was a prominent barrier:

This happens to me frequently throughout the day, I catch myself doing it automatically, again and again […] without even realizing I’m doing it.

[Participant 11, position 14]

However, participants also reported that identifying and implementing functional substitutes facilitated smoking reduction or cessation:

So what you should do instead, for example, go for a walk or something like that. I found it really difficult to find things, including this reward. But in the end, I really noticed that it helped me. So, that was really good.

[Participant 4, position 42]

Addressing habitual aspects of smoking by modifying environmental cues also emerged as a facilitative strategy to disrupt automatic smoking patterns:

Well, at least I now have several smoke-free zones in my flat. That wasn’t the case before.

[Participant 11, position 14]

However, some participants reported anxiety regarding the loss of smoking as a primary emotion regulation strategy, fearing that some new coping strategies might also turn into maladaptive behaviors:

So with smoking, it was very, very often a way for me to regulate my emotions, regulate stress and [...] I was just worried [...] okay, I have to replace it with something else now so that I can cope. And I was worried that it might somehow turn into something else.

[Participant 1, position 30]

#### Subtheme 2: Motivation

Another central component frequently cited as a particularly important factor in participants’ smoking cessation trajectories was motivation, which was described as a dynamic and context-sensitive process. At treatment entry, participants retrospectively described varying levels of motivation, ranging from strong readiness to quit smoking to considerable difficulties engaging with the cessation process. Furthermore, participants frequently articulated the belief that intrinsic motivation and readiness to change were essential for cessation success (“It’s just that you can’t convince someone to stop if they don’t really want to” [Participant 3, position 28]). Importantly, motivation was described as malleable over the course of the treatment. Participants reported increases in intrinsic motivation due to the motivation analysis conducted in the second session, which was described as strengthening deliberate and autonomous decision-making:

What I found very nice, was […], that it was always made clear to you that, in the end, there are also advantages, […] with smoking, that it’s not only negative and that you decide, weigh up the pros and cons […], and that is a realistic view of the situation, that it’s also a decision in a way and that we can make it for ourselves. And […] that really helped me. […] It had a big impact on my motivation.

[Participant 1, position 12]

Beyond formal treatment components, sustained abstinence itself was described as intrinsically motivating:

So, nothing [motivated me] at first, I’d say, but then over time, okay, now you’ve already made it through a week, two weeks.

[Participant 6, position 28]

Information regarding the anticipated mental health benefits of smoking cessation, as well as experiencing them, was also reported as particularly encouraging:

These positive effects, which I have been informed will occur after a while, […] I have already noticed them happening and am experiencing them. And I must also say that I would not want to miss out on these positive effects.

[Participant 3, position 38]

Despite sustained or increasing motivation during the active treatment phase, many participants reported motivational fluctuations following the end of the structured cessation sessions due to the perceived loss of interpersonal accountability and support:

This personal connection, which you then also have with your colleague or with you. That was probably the deciding factor for me. And when that disappeared, my motivation also disappeared a little bit.

[Participant 10, position 10]

However, despite fluctuations in motivation following treatment completion, a long-term intention to quit often remained (“I still have that goal, of course” [Participant 11, position 6]).

#### Subtheme 3: Social and Contextual Influences

Participants frequently emphasized the role of their social networks and immediate contexts, which both facilitated and impeded cessation success. In particular, several participants identified their smoking-normative social environment as a significant barrier to cessation:

Yes, many of my friends are smokers. Indeed. And that just makes you more and more motivated to start again or continue smoking.

[Participant 2, position 40]

These impediments were often attributed to concrete social processes, such as the presence of situational cues, which reinforced smoking or experiencing social pressure to accompany smoking peers to designated smoking areas:

[...] But there was still pressure, pressure from the others, who would say, ‘Hey, I’m going for a smoke, do you want to come with me, even if it’s just to get some fresh air?’ And at some point, it just turned into smoking again.

[Participant 2, position 42]

Beyond direct social pressure, smoking was also perceived as fulfilling distinct social functions that reinforced cessation barriers. Participants described smoking breaks as structured social intervals that facilitated interaction and provided temporal orientation within social settings:

Of course. You can go outside, then you’re standing there. What do you do with your hands now? […] How long do I stay outside now […], the social bonding [was gone]. The cigarette.

[Participant 8, position 93]

Notably, despite describing their smoking-heavy environments as challenging, some participants simultaneously reported receiving encouragement from smoking individuals for their cessation attempts:

They all thought it was great. […] let’s say almost ten of the closest ones […] so almost all of them [smoke].

[Participant 8, positions 83-85]

In addition, participants attributed a substantial portion of their cessation success to supportive social networks, as encouragement and understanding were described as critical resources, particularly during high-risk situations:

Yes, I’ve talked about it with my best friend quite a few times. […] When I had a strong craving for a cigarette, I told her about it and explained why I had this craving and what triggered it. She was very supportive.

[Participant 3, position 72]

Lastly, participants identified the disclosure of their cessation attempt to their social network as a facilitating factor. Although initially perceived as pressure-inducing, disclosure was retrospectively framed as increasing accountability and commitment:

With some people, I don’t think they necessarily involve their social circle, and I have to say, when I disclosed that, at first, I was like, okay, that’s a lot of pressure, but if I don’t manage it now, it feels stupid, but then you know, okay, I think it’s fine if you just try to commit at the beginning.

[Participant 1, position 56]

#### Subtheme 4: Indication

Lastly, participants expressed heterogeneous perceptions regarding the appropriateness of the smoking cessation treatment for individuals with comorbid mental disorders. Some participants considered the intervention broadly suitable (“I think it’s suitable for anyone who wants to quit but can’t manage it*”* [Participant 10, position 51]). In contrast, a subset of participants expressed skepticism regarding its applicability, citing high effort, potential exacerbation of symptoms, or interference with ongoing therapy:

Yes, it depends on how the person behaves in my eyes. Of course, if they have problems such that […] their mental state deteriorates further, then I don’t know if it’s the right thing to do at that moment.

[Participant 5, position 44]

It can definitely lead to other relapses, whether it’s self-harming behavior or slipping into an eating disorder.

[Participant 2, position 66]

I don’t know whether it makes sense to try to quit smoking right in the middle of therapy or at the very beginning of therapy. It could well hinder the positive progress of the therapy.

[Participant 2, position 60]

Skepticism was occasionally reinforced by perceived professional caution, with participants referencing health care providers’ reluctance to recommend cessation under high-stress conditions:

And I also know that many doctors do not approve of this at the moment, that perhaps at a time when you are already under a lot of stress, you are adding yet another burden to yourself because of it.

[Participant 4, position 44]

Notably, participants’ concerns contrasted with their own reported outcomes: despite skepticism about the treatment’s suitability for smokers with mental disorders, the same individuals generally described beneficial mental health effects and only short-lived negative consequences following cessation.

Overall, participants identified multiple facilitators and barriers to cessation, including the specific functions and automaticity of smoking, motivation, social networks, contextual influences, and treatment suitability for those with comorbid mental disorders. Taken together, these accounts suggest that cessation processes are closely intertwined with how participants experience and make use of the support provided during treatment, as well as how this support fits with their individual needs and circumstances.

### Theme 3: Treatment Engagement as Contextually and Motivationally Contingent

Participants offered diverse evaluations of the smoking cessation treatment, reflecting that engagement with and benefit from the intervention was shaped by individual motivational states, contextual circumstances, and the degree to which treatment components aligned with personal needs. Rather than uniform experiences, participants described a range of responses to specific treatment modules and procedures.

#### Subtheme 1: Perceptions of the Manual-Based Smoking Cessation Intervention

Participants generally expressed favorable perceptions of the manual-based smoking cessation treatment, highlighting its structured modules, practical strategies, and the focus on alternative behaviors to replace smoking:

Well, I found it very helpful to write down the situations in which I typically smoke and the reasons why I smoke in those situations. And then, um, writing down a counterproposal, so to speak […] because I thought it was good to first become aware of the issue, but also to have a counterproposal to the bad behavior […].

[Participant 7, position 16]

Similarly, preparation for high-risk situations was also described as beneficial, as participants were provided with concrete strategies for managing acute craving:

Specifically, what helped me, what comes to mind right now, was a list that I had put up on my desk. So, what I can do when I suddenly have a craving, as an emergency intervention or crisis plan, that helped me a lot

[Participant 1, position 10]

Conversely, the applied CO-measure elicited both motivational effects (“[…] seeing a lower [CO] number can definitely motivate you” [Participant 8, position 29])*,* as well as a feeling of being controlled:

Well, I always felt a bit uncomfortable doing that puff test at the beginning, but that was more, because you are made aware [of the CO-value] and you know someone is checking it.

[Participant 7, position 20]

Beyond the perceptions regarding specific modules and components of the cessation treatment, participants also delineated factors they perceived as beneficial in general. For instance, the broader psychotherapeutic context of the intervention was frequently cited as a key contributor to its efficacy:

So, these conversations helped me a lot, a few interventions or, for example, the one about the pros and cons. I had already tried that out for myself, but it was still something different to get input from a third person.

[Participant 1, position 2]

Additionally, regular, structured appointments were perceived as reinforcing commitment, in part due to the motivational influence of accountability within the therapeutic relationship.

Because I always had these little goals in mind. These weeks. Appointments. […] It helped a lot that it was regular […].

[Participant 9, position 20]

#### Subtheme 2: Perceptions on the Conjunctive App-Treatment

As study participation additionally involved the prescription of an application designed to facilitate smoking cessation, participants offered a range of comments pertaining to this application. Participants’ engagement with the application varied considerably. Some participants explicitly attributed a substantial portion of their motivation and cessation success to the application:

I have to say that the app really helped me. I really enjoyed the conversations with Ms. X [therapist], but it was the app that gave me the final push. Honestly.

[Participant 4, position 16]

Conversely, other participants reported limited or no use of the application, indicating that they did not perceive a sustained need for its support (“Because I just did not need it” [Participant 5, position 46‐48]) or expressing doubts regarding its efficacy:

Yes, but I have to say […] when I want to smoke, I smoke. It’s [the app] not something that motivates me or makes me think twice.

[Participant 3, position 54]

This heterogeneity in perceived utility, ranging from the app constituting a decisive treatment component to being perceived as unnecessary or ill-fitting, points to individual differences in receptiveness to digital treatment components rather than a uniform response to the app itself. For some participants, this reduced receptiveness extended beyond the specific application to a more general reluctance toward mobile applications:

Because I hardly ever use my smartphone. And I don’t have a calendar or anything like that that I use. […]. Somehow, I’m a bit averse to it. […] There’s no reason, except that I didn’t want to.

[Participant 10, position 63]

#### Subtheme 3: Perceived Mismatch Between Treatment Structure and Individual Needs

Beyond their evaluation of specific treatment components, participants also articulated a range of recommendations for optimizing the cessation treatment. Several of these recommendations pointed toward a recurring tension between the standardized structure of the intervention and participants’ individually fluctuating needs, particularly with respect to treatment timing and intensity. For instance, participants suggested that the treatment could be adapted to better accommodate periods of heightened psychological strain:

Perhaps to help these setbacks, in the psychological wellbeing, to not cause one to lose focus on the treatment itself.

[Participant 12, position 36]

Several participants emphasized the importance of flexible scheduling, noting discrepancies between the standardized frequency of sessions and their personal needs:

I remember I had four or five conversations, I had already been there for a relatively long time, and consistent, and, […] I realized that it wasn’t really necessary for me. […] And at the beginning, I might have found a little more, perhaps even more frequent conversations, to be helpful.

[Participant 1, position 18]

Similarly, participants also recommended generally increasing the intensity and duration of treatment appointments, specifically regarding the time available for conversations to enable discussing certain topics more in depth:

I would have liked to have had a little more time here and there during the conversations, than what was available, yes.

[Participant 11, position 36]

A subset of participants highlighted the potential values of tailored follow-up appointments for individuals concerned about acute relapse:

And I think it can be very individual. It may also be that after two or three months, you say, okay, I’m now really afraid of relapsing or something, […].

[Participant 1, position 18]

Taken together, these accounts suggest that the standardized format of guideline-conform treatment may itself introduce distinct challenges for individuals with comorbid mental disorders, particularly regarding the timing, intensity, and individualization of support. Beyond the structure and delivery of the treatment itself, participants also identified the need for enhanced awareness and outreach, noting that promotion of cessation programs, particularly for individuals with mental comorbidities, was insufficient, but could increase uptake among those who might otherwise remain unaware of available resources:

I think a little marketing is needed so that people find out about it, right? Because I didn’t know anything about it either. […] I would never have come up with this idea on my own.

[Participant 10, position 26]

In sum, participants had a broad spectrum of perceptions of the smoking cessation treatment in this overarching theme. Overall, experiences were predominantly positive, particularly with respect to the manual-based intervention and the psychotherapeutic context. The adjunctive app elicited more mixed responses, particularly regarding its utility. Nonetheless, participants frequently characterized study participation as akin to therapeutic engagement. Importantly, they provided a range of recommendations for optimizing the intervention, emphasizing the need for greater individualization to accommodate personal circumstances and psychological states, as well as intensified support during critical phases of cessation.

## Discussion

### Principal Findings

This study explored the experiences of individuals with comorbid mental disorders participating in a manual-based, guideline-conform smoking cessation treatment, using semistructured interviews to capture nuanced insights into treatment engagement, perceived challenges, and facilitating factors. Three overarching themes were identified: cessation as a multidimensional, processual experience; smoking as a functionally embedded practice shaped by motivation, social networks, and contextual influences; and treatment engagement as contextually and motivationally contingent. Taken together, these findings suggest that guideline-conform smoking cessation treatment is feasible and largely well-received by individuals with comorbid mental disorders, while also highlighting specific areas for intervention optimization. The following sections integrate findings across themes to illuminate the central processes shaping smoking cessation in this population.

### Comparison to Prior Work and Implications

#### Smoking Cessation Outcomes as Processual and Nonlinear

Participants’ accounts challenge binary notions of cessation success versus failure. This nonlinear pattern of abstinence, reduction, relapse, and shifts in use is in line with process-oriented models of behavior change, such as the transtheoretical model [50,51], which conceptualizes relapse as an integral component rather than an endpoint. From a qualitative perspective, participants’ narratives suggest that subjective success was in several cases defined through increased awareness, perceived agency, or harm reduction rather than sustained abstinence alone. These findings underscore the importance of distinguishing between clinically defined outcomes and experientially meaningful change, particularly in populations with complex psychosocial needs. Sustained abstinence remains the primary therapeutic goal, as reflected in current treatment guidelines [7]; however, for individuals with comorbid mental disorders who struggle to achieve full abstinence, smoking reduction and nicotine substitution may serve as pragmatic intermediate steps, potentially easing craving and supporting eventual quit attempts [52,53], though evidence that reduction independently delivers long-term health benefits without eventual abstinence remains limited [52]. Accordingly, the present findings suggest that treatment frameworks for this population may benefit from complementing abstinence-focused goals with harm reduction perspectives that recognize reduction and substitution as pragmatically valuable steps within a broader cessation trajectory.

#### Mental Well-Being: Self-Efficacy, Distress, and Adaptive Meaning-Making

Changes in smoking behavior were closely intertwined with changes in mental well-being. Improvements in mood, energy, and psychological stability following abstinence or reduction were commonly attributed to feelings of pride, accomplishment, and mastery, closely mirroring Bandura’s [54] concept of self-efficacy: successful cessation attempts can be understood as “mastery experiences” that strengthened beliefs in one’s capacity for self-regulation and coping. In this sense, smoking cessation extended beyond smoking behavior change to become an identity-relevant experience that reinforced agency and self-confidence.

At the same time, early-abstinence strain (withdrawal, heightened negative affect) was typically framed as intense but time-limited, and relapse-related guilt or disappointment as transient and proportionate; neither escalated into broader crises nor affected core mental health symptoms, consistent with evidence that cessation does not generally worsen, but may improve mental health outcomes among individuals with mental disorders [33,34]. Rather, participants’ accounts suggest that distress appeared cognitively integrated into a broader narrative of change, enabling continued engagement despite setbacks, while increases in self-efficacy and perceived agency suggested positive downstream effects on the psychotherapeutic process itself, a hypothesis further examined in the embedded RCT by Sünkel et al [43]. These findings do not support the assumption that cessation or relapse inherently destabilizes individuals with mental disorders; concerns about severe emotional destabilization should therefore not be considered a general contraindication to recommending smoking cessation in this population.

#### Smoking as a Functional and Embedded Coping Practice

A central barrier to cessation concerned the perceived functionality of smoking, which served emotion regulation, stress relief, cognitive activation, time structuring, and social facilitation—consistent with negative reinforcement and affect regulation models of addiction, which emphasize smoking’s role in alleviating aversive internal states [55]. Importantly, several participants found it helpful when these functions were explicitly acknowledged within the intervention, as this validation supported autonomous decision-making rather than framing smoking solely as maladaptive, resonating with motivational interviewing’s nonjudgmental exploration of ambivalence and respect for autonomy [56,57]. Participants also highlighted interventions aimed at developing concrete alternatives for coping and stress management (eg, progressive muscle relaxation) as particularly helpful, underscoring the importance of identifying and practicing situation-specific alternatives. For participants with mental comorbidity, concerns about losing a primary coping mechanism were especially salient and often persisted despite psychoeducation about the benefits of cessation, pointing to the psychological complexity of cessation in this population and the need for more tailored interventions. At the same time, the strong habitual and automatic embedding of smoking behaviors represents a nontrivial barrier to change, suggesting that cessation may require not only reflective and verbal interventions but, in some cases, complementary approaches that directly target automatic action tendencies. Cognitive bias modification, which aims to retrain maladaptive approach biases at a motoric level, has been proposed and empirically examined in smoking cessation [58-60] and may therefore represent a useful adjunct to interventions primarily focused on reflective decision-making.

#### Motivation as Dynamic, Relational, and Context-Sensitive

Motivation emerged as highly fluctuating across cessation trajectories. Although intrinsic motivation and readiness to change were frequently described as important at treatment entry, participants’ accounts clearly indicate that motivation was continuously shaped and reshaped throughout the cessation process. Intervention components such as motivational analyses, CO feedback, and early abstinence successes were experienced as reinforcing commitment and engagement, underscoring the situational and processual nature of motivation as a state rather than a stable trait. This interpretation is consistent with empirical evidence suggesting that baseline, trait-like motivation alone is not a reliable predictor of smoking cessation success. For example, Ussher et al [61] found that the strength of motivation to quit assessed prior to treatment did not predict abstinence among treatment-seeking smokers, yet dependence severity did. At the same time, motivational processes unfolding during the quit attempt itself may be more relevant for understanding lapse and relapse. Motivation appears to serve different functions at different stages of behavior change: while motivation measures such as the Motivation To Stop Scale show strong predictive validity for initiating quit attempts over several months [62], they may be less informative for predicting sustained abstinence. This distinction closely mirrors participants’ narratives, in which motivation was described as sufficient to initiate a quit attempt but vulnerable to contextual influences once abstinence was underway.

Conceptualizing motivation as a dynamic state rather than a stable trait also provides a natural link to self-determination theory [63], which has also received empirical support in the context of smoking cessation [64]. Rather than assuming enduring motivational dispositions, the theory emphasizes the situational and context-dependent regulation of motivation as a function of momentary satisfaction or frustration of basic psychological needs for autonomy, competence, and relatedness. In this sense, self-determination theory offers a framework for understanding why motivation during smoking cessation is experienced as unstable yet responsive to intervention components. Consistent with this perspective, participants’ accounts highlighted the importance of being able to make autonomous decisions, experiencing competence through early successes, and feeling supported by others. In this context, Niemiec and colleagues’ [64] concept of motivational attunement is particularly informative. Attending to daily motivational fluctuations and responding with timely, need-supportive interventions aligns closely with participants’ experiences. In participants’ accounts, feeling heard and supported coincided with greater engagement and readiness for change. The motivational decline following the end of structured treatment highlights the importance of ongoing relational support and accountability (eg, booster sessions). Taken together, these findings suggest that future interventions may benefit from adopting motivation-sensitive designs in which the intensity, timing, and form of support are flexibly adjusted to individuals’ momentary motivational states (eg, ecological momentary assessment approaches as described by Shiffman [65]).

#### Social and Contextual Embeddedness of Smoking Cessation

Smoking cessation was embedded within broader social and contextual environments, with social networks acting as both facilitators of and barriers to change. In this study, significant others who smoked were predominantly experienced as relapse risk factors—observing friends or family smoke increased urges and, in some cases, reinforced participants’ own smoking—while supportive relationships provided emotional and practical assistance during acute craving. Even with explicit encouragement to quit, smoking-normative environments and shared smoking rituals often undermined cessation efforts, illustrating social context’s complex, sometimes ambivalent influence on behavior change. These findings align with social practice theory [66], which frames smoking as a practice organizing interaction, time, and relational proximity. From this perspective, cessation requires not only individual self-regulation but also the renegotiation of social roles, routines, and meanings. Contextual factors such as timing, stress exposure, and entrenched habits further shaped participants’ experiences, highlighting the interplay between individual agency and situational constraints.

Clinically, these findings suggest that smoking cessation interventions may benefit from explicitly addressing the social embeddedness of smoking. Supporting individuals in navigating high-risk social contexts, renegotiating shared smoking rituals, and mobilizing supportive relationships in ways that foster relatedness without exerting pressure may help reduce the social costs of cessation while strengthening motivation and persistence. In other areas of addiction treatment and psychotherapy, the inclusion of partners or family members is a common practice and has been shown to improve engagement and treatment outcomes [67].

#### Indication and Clinical Caution in the Context of Comorbid Mental Disorders

A particularly salient finding concerns participants’ heterogeneous views on the indication of cessation treatment for individuals with comorbid mental disorders, ranging from strong endorsement to pronounced skepticism about its feasibility and safety during periods of heightened vulnerability, reflecting broader clinical and cultural narratives within mental health care. Concerns primarily centered on the perceived risk that cessation could exacerbate psychiatric symptoms, destabilize emotion regulation, or interfere with ongoing psychotherapeutic processes. Several participants explicitly referenced assumptions commonly attributed to health care professionals, such as the belief that smoking cessation constitutes an additional burden during acute distress or early treatment phases. Notably, these concerns persisted despite participants’ own experiences frequently contradicting such assumptions, as most reported improvements in mental well-being or only short-lived adverse effects following cessation attempts. This discrepancy highlights a critical tension between anticipated risk and experienced outcomes. The persistence of concerns despite predominantly disconfirming personal experiences can be understood in light of the ViolEx model of expectation maintenance and change [68,69]. According to this framework, expectations often remain stable even after violation due to cognitive immunization processes, in which discrepant experiences are reinterpreted as exceptions rather than prompting accommodation. In the present data, immunization can be interpreted in person-bound attributions (eg, cessation being feasible “for me, but not for more severely ill individuals”) and temporal qualifications (eg, cessation being possible only because it was “the right time”). Such interpretations preserve the overarching expectation that smoking cessation poses a risk during mental health treatment, despite experiential evidence to the contrary. Extensions of the ViolEx model [70,71] emphasize that expectation change requires not only experiential violation but also the explicit reduction of immunizing explanations. From this perspective, the findings suggest that integrating expectation-focused reflection into smoking cessation interventions may be beneficial for modifying anticipatory risk beliefs and facilitating broader acceptance of cessation within psychotherapeutic care. While participants acknowledged that cessation can be demanding, their narratives largely challenge the notion that smoking cessation is inherently contraindicated for individuals with mental health difficulties. Instead, the findings suggest that perceived risks may stem less from cessation itself than from insufficient support, suboptimal timing, or a lack of contextual sensitivity in treatment delivery.

These findings align with current clinical guidelines recommending that individuals with comorbid mental disorders should receive smoking cessation support on equal terms with the general population [7]. However, smoking continues to be framed by many health care professionals as a “lifestyle issue” rather than a life-threatening condition, and qualitative research in psychiatric care settings similarly documents persistent misconceptions among practitioners, including beliefs that cessation may harm patients with mental disorders [32]. The present study extends this literature by showing that such misconceptions are experienced by patients themselves, even when their own cessation attempts led to positive or stabilizing mental health outcomes [33,34].

### Limitations

Consistent with reflexive thematic analysis, the presented themes represent one interpretative account shaped by the researchers’ analytical lens, the study’s exploratory aims, and the interactional context of the interviews. Both analysts had completed training in the intervention, which may have introduced familiarity-related assumptions even as it enhanced sensitivity to participants’ experiences; alternative interpretations of the data therefore remain possible. The analysis was conducted inductively and themes were grounded iteratively in participants’ accounts; however, readers should bear in mind that all qualitative findings are necessarily perspectival and that alternative interpretations of the data remain possible. In addition, the study’s findings must be considered in light of the small sample with most participants having had a primary diagnosis of depression, with only single cases of social phobia and bulimia nervosa, limiting the extent to which the findings can be generalized to individuals with other mental health conditions. It therefore remains unclear to what degree the identified processes and implications apply to smokers with different psychiatric diagnoses or symptom profiles. Reliance on retrospective self-reports may also have introduced recall biases. Furthermore, it is worth noting that several participants reflected on the dual nature of study participation, describing the research context as both evaluative and therapeutically meaningful. While this observation did not constitute a primary analytic finding directly addressing the study’s aims, it suggests that participation in research embedded within a treatment context may itself carry therapeutic valence, a consideration relevant to the interpretation of findings and the design of future embedded qualitative studies.

### Conclusions

Taken together, the findings of this study underscore several clinically relevant implications. First, the present findings suggest that smoking cessation should be conceptualized and communicated as a dynamic, nonlinear process rather than a binary outcome. Clinicians may benefit from validating partial reductions, increased awareness, and temporary setbacks as meaningful steps within a broader change trajectory, particularly for individuals with comorbid mental health conditions.

Second, interventions should explicitly address the functional role of smoking in emotion regulation, stress management, and social interaction. Supporting individuals in developing alternative coping strategies, while acknowledging fears related to losing a primary coping mechanism, may be especially important in vulnerable populations. Such support should be delivered in an autonomy-supportive manner that fosters competence and relatedness, consistent with the self-determination theory [63].

Third, future research should examine how motivational fluctuations unfold in everyday contexts and how interventions can be adaptively tailored to them. Participants wanted greater flexibility in treatment structure and timing, for example, adjusting session frequency to current motivation, extending treatment with lower-frequency maintenance sessions, and involving significant others where appropriate. Longitudinal, mixed method designs may be especially valuable for capturing this dynamic interplay over time.

Finally, the findings highlight the need to deconstruct persistent misconceptions surrounding smoking cessation in mental health care, consistent with the ViolEx-based immunization processes [68,69] discussed above. This warrants targeted psychoeducation and expectation-focused reflection for patients and practitioners alike, challenging the assumption that cessation worsens mental health and emphasizing its potential psychological benefits. Actively facilitating expectation change may help reduce unnecessary clinical caution and support more consistent and confident integration of smoking cessation into psychotherapeutic care.

## Supplementary material

10.2196/97001 Multimedia Appendix 1 Interview guide.

10.2196/97001 Checklist 1 COREQ checklist.

## Abbreviations

CBT: cognitive behavioral therapy
CCI: Client Change Interview
CO: carbon monoxide
COREQ: Consolidated Criteria for Reporting Qualitative Research
*ICD-10*: *International Statistical Classification of Diseases, Tenth Revision*
RCT: randomized controlled trial
