# Supplementary material for: Standardized Manual-Based Smoking Cessation Treatment in Patients With Comorbid Mental Disorders: Qualitative Interview Study
Source: JMIR Form Res. 2026 Sep 23;10:e97001. doi: 10.2196/97001 (PMC13600660; doi:10.2196/97001)
Supplement: Supplementary file 2 [file formative-v10-e97001-s001.docx]

**Interview guide in the original German version**

*Interviewer: „Es wird einen 2. Teil geben, in dem ich noch einmal genauer auf einzelne Aspekte eingehen werde, aber zunächst würde mich interessieren, wie Sie die Behandlung allgemein erfahren haben.“*

1. *Generelle Erfahrung der Behandlung:*
   a. Wie haben Sie die Behandlung erfahren?
   b. Wie hat es sich angefühlt, daran teilzunehmen?
2. *Veränderungen:*
   a. Gibt es Veränderungen an Ihnen, die Ihnen seit Behandlungsbeginn aufgefallen sind?
3. *Attribution:*
   a. Allgemein gesehen, wem oder was schreiben Sie diese Veränderungen zu?
   b. In anderen Worten, wodurch kamen diese Veränderungen zustande?
4. *Hilfreiche Aspekte/Inhalte*:
   a. Gab es Aspekte/Inhalte der Behandlung, die Sie als hilfreich erfahren haben?
   b. Welcher Aspekt/Inhalt war der hilfreichste?
   c. Was daran fanden Sie besonders hilfreich?
   d. Woran, glauben Sie, lag das?
5. *Hindernde Aspekte/Inhalte:*
   a. Gab es Aspekte/Inhalte, die nicht hilfreich, hindernd, negativ oder enttäuschend für Sie waren?
   b. Woran glauben Sie lag das, dass diese Aspekte nicht hilfreich waren?
   c. Was hätten Sie sich stattdessen gewünscht?
6. *Schwierige, aber trotzdem hilfreiche Aspekte/Inhalte:*
   a. Gab es Aspekte/Inhalte, die schwierig oder unangenehm waren, aber trotzdem „ok“ oder hilfreich?
7. *Fehlende Aspekte/Inhalte:*
   a. Hat etwas in der Behandlung gefehlt?
   b. Was sind mögliche Hürden, die diese Rauchentwöhnungstherapie noch überwinden müsste, um mehr Zulauf zu gewinnen?
8. *Motivation:*
   a. Wie leicht fiel es Ihnen, sich für die Rauchentwöhnung zu motivieren?
   b. Stand etwas Ihrer Motivation im Weg?
   c. Was hat Sie motiviert? / Was hat Ihre Motivation behindert?
9. *Empfehlungen:*
   a. Hätten Sie Anregungen oder Empfehlungen, um die Rauchentwöhnungstherapie ansprechender zu gestalten?
   b. Würden Sie die Behandlung weiterempfehlen?
   c. Haben Sie Veränderungen in Ihrem Rauchverhalten festgestellt? Denken Sie, dass diese Behandlung eine gute Alternative zu anderen Therapien zum Rauchstopp darstellt?
   d. Haben Sie Veränderungen in Ihrem psychischen Wohlbefinden wahrgenommen?
   e. Wie würden Sie die Eignung der Rauchentwöhnungstherapie bei Personen mit einer psychischen Störung einschätzen?
   f. Sehen Sie hier Schwierigkeiten?
10. *Hilfesetting*
    a. Haben Sie die verschriebene App zur Rauchentwöhnung genutzt?
    b. Haben Sie Nikotinersatzpräparate verwendet?
    c. Haben Sie Nikotin in anderer Form zu sich genommen (Pouches, Vaping, …)?
11. *Nicht intendierte Folgen und Einflüsse des sozialen Umfelds:*
    a. Hatte die Behandlung Folgen, die Sie nicht erwartet haben?
    b. Haben Sie positive Auswirkungen der Behandlung auf bestimmte Lebensbereiche bemerkt?
    c. Haben Sie negative Auswirkungen der Behandlung auf bestimmte Lebensbereiche bemerkt?
    d. Wie hat Ihr soziales Umfeld auf Ihre Behandlung und die Veränderungen, die sich womöglich durch Ihre Teilnahme ergeben haben, reagiert?
    e. Haben Sie Unterstützung aus Ihrem sozialen Umfeld erfahren?
    f. Haben Sie Veränderungen in Ihrem sozialen Umfeld bemerkt?
12. *Forschung:*
    a. Wie war es für Sie, an einer Studie im Rahmen von Forschung teilzunehmen?

**Interview guide translated to English**

*Interviewer: “There will be a second part in which I will go into more detail on specific aspects, but first I’d like to know how you experienced the treatment in general.”*

1. Overall experience with the treatment:
   a. How did you experience the treatment?
   b. How did it feel to participate in it?
2. Changes:
   a. Have you noticed any changes in yourself since the treatment began?
3. Attribution:
   a. Generally speaking, to whom or what do you attribute these changes?
   b. In other words, what caused these changes?
4. Helpful aspects/content:
   a. Were there any aspects or elements of the treatment that you found helpful?
   b. Which aspect or element was the most helpful?
   c. What did you find particularly helpful about it?
   d. Why do you think that was?
5. Hindrances/Issues:
   a. Were there any aspects or content that you found unhelpful, hindering, negative, or disappointing?
   b. Why do you think these aspects were unhelpful?
   c. What would you have preferred instead?
6. Difficult but still helpful aspects/content:
   a. Were there any aspects or content that were difficult or unpleasant but still “okay” or helpful?
7. Missing aspects/content:
   a. Was anything missing from the treatment?
   b. What are some potential hurdles this smoking cessation therapy would still need to overcome to gain more popularity?
8. Motivation:
   a. How easy was it for you to motivate yourself to quit smoking?
   b. Was there anything that stood in the way of your motivation?
   c. What motivated you? / What hindered your motivation?
9. Recommendations:
   a. Do you have any suggestions or recommendations for making smoking cessation therapy more appealing?
   b. Would you recommend this treatment to others?
   c. Have you noticed any changes in your smoking behavior? Do you think this treatment is a good alternative to other smoking cessation therapies?
   d. Have you noticed any changes in your mental well-being?
   e. How would you rate the suitability of smoking cessation therapy for people with a mental disorder? Do you see any difficulties here?
10. Support Setting
    a. Did you use the prescribed smoking cessation app?
    b. Did you use nicotine replacement products?
    c. Did you consume nicotine in other forms (pouches, vaping, …)?
11. Unintended consequences and influences of the social environment:
    a. Did the treatment have consequences you did not expect?
    b. Have you noticed positive effects of the treatment on certain areas of your life?
    c. Have you noticed negative effects of the treatment on certain areas of your life?
    d. How did your social environment react to your treatment and the changes that may have resulted from your participation?
    e. Did you receive support from your social environment?
    f. Did you notice any changes in your social environment?
12. Research:
    a. What was it like for you to participate in a research study?
